# Dietary Habits and Lifestyle Behaviors of Saudi Residents during the COVID-19 Pandemic: A Cross-Sectional Study

**DOI:** 10.3390/ijerph19137659

**Published:** 2022-06-23

**Authors:** Taqwa Bushnaq, Reham M. Algheshairy, Mona S. Almujaydil, Asma Al Malki, Hend F. Alharbi, Hassan Barakat

**Affiliations:** 1 Department of Food Science and Nutrition, College of Science, Taif University, P.O. Box 11099, Taif 21944, Saudi Arabia; tabushnaq@tu.edu.sa (T.B.); asma@tu.edu.sa (A.A.M.); 2 Department of Food Science and Human Nutrition, College of Agriculture and Veterinary Medicine, Qassim University, Buraydah 51452, Saudi Arabia; m.almujaydil@qu.edu.sa (M.S.A.); hf.alharbi@qu.edu.sa (H.F.A.); 3 Department of Food Technology, Faculty of Agriculture, Benha University, Moshtohor 13736, Egypt

**Keywords:** COVID-19, coronavirus, eating habits, lifestyle behaviors, cross-sectional

## Abstract

Coronavirus disease 2019 (COVID-19) has continued to spread rapidly worldwide, forcing countries to enact lockdowns and strict social-distancing measures that affect individual and population health. This study investigates the changes in dietary habits and lifestyle behaviors among Saudi residents during the COVID-19 pandemic. A cross-sectional survey was conducted on 786 participants, with a mean age of 30 years. The questionnaire was administered online and sent via social media applications. Dietary habits, physical activity, TV-watching time, sleep patterns, stressors, and socio-demographic data were evaluated. Among the survey respondents, there was an increase in weight gain, food consumption, and decreased physical activity. Respondents who gained weight consumed more snacks, ate larger food portions, and engaged in less physical activity. Furthermore, a high fish, water, and supplements intake was noticed. Although respondents’ intake of fruits and vegetables was low, most individuals reported a negatively changed intake. However, most respondents reported increased consumption of lean meat and sweet foods. Observing changes in dietary and lifestyle habits during the lockdown period may help elaborate on the pandemic’s consequences for population health and assist in formulating and implementing policies for future closures, while supporting vulnerable groups.

## 1. Introduction

On 11 April 2020, the World Health Organization (WHO) declared coronavirus (COVID-19) to be a pandemic [1]. COVID-19 research is a fast-expanding subject globally, and the single most-successful approach was social distancing, which can reduce infection rates by up to 92% [2]. Most countries followed similar mitigation approaches such as area lockdowns, curfews, and travel suspension. After the first confirmed case, the Saudi government announced a series of drastic measures to stem the spread of the virus, including banning all travel in and out of the Qatif governorate between 8 March and 6 April 2020 and enforcing a 24 h curfew in major cities, with only essential travel allowed between 6 a.m. and 3 p.m. [3]. The forceful attempts to prevent the virus from spreading have contributed to public dread, anxiety, and/or despair, which are often overlooked during crisis and pandemic management [4]. The effects of such long-term restrictions on the population were mixed [5]. The goals were to reduce the spread of infection and mortality by COVID-19. Still, they also caused a drastic shift in people’s lives, potentially affecting the community’s health and well-being.

Understanding the lifestyle consequences of the COVID-19 infection-reduction methods may aid in making well-informed decisions on the appropriate strategies to use in similar future scenarios [6]. Several publications show that negative factors such as unhealthy habits, economic crises, stress, and loneliness can lead to negative lifestyles [7,8].

The individuals in Saudi Arabia (SA) experienced extraordinary behavioral changes in all parts of their lifestyle, with some positive effects on smoking and food habits [9]. Unemployment, physical inactivity, sleep difficulties, social isolation, and excessive weight gain were negative changes [9]. Ashgar [10] reported that nutritional behaviors were positively correlated with sex, age, and having a job before and/or after the pandemic, which were negatively correlated with the number of people in the household. Moreover, interpersonal relationships increased with age and years of education. Interpersonal relationships and stress management decreased among those who reported that the pandemic had negatively affected their income. It is concluded that COVID-19 factors are associated with Saudi adults’ adoption of health-promoting behavior. Indeed, Alhazmi et al. [11] showed sufficient knowledge, attitude, and practice toward COVID-19 in SA. The educational level was a dominant influencing factor for knowledge, attitude, and practice.

During the pandemic, the lockdown imposed in SA modestly but significantly impacted several citizens and residents’ physical activity and dietary behaviors in an unhealthy way [12]. Similarly, Leila et al. [13] indicated that individuals experienced negative lifestyle changes, unbalanced food choices, reduction in physical activity, and psychological problems during the COVID-19 pandemic. A cross-sectional study suggests that the COVID-19 pandemic triggered unhealthy lifestyle changes, physical inactivity, and psychological issues in the Middle East and North Africa. A substantial need to increase awareness regarding healthy nutritional habits, general safety measures, the importance of home-based physical activity, and stress-relief mechanisms were highly recommended [14]. Consequently, numerous scholars have raised concerns regarding the effects of precautionary home quarantine on individuals’ physical and mental health, along with a possible worsening of eating habits and low physical activity during home quarantine. In addition, poor food choices were reported in other countries during home-quarantine periods [8]. Poor diet and low physical activity have been linked to numerous non-communicable and communicable diseases [7,15,16,17]. It has been suggested that these changes in eating and lifestyle habits can be attributed to reduced access to shops due to lockdowns, a lack of variety in food types, and poor snack choices [7].

During pandemics, it is critical to consider multiple areas of human health, such as dietary habits and the quality and quantity of foods, as these factors play roles in the immune system and general health. Therefore, this study investigates the effect of precautionary full home quarantines on Saudi residents regarding eating habits and lifestyle behaviors.

## 2. Materials and Methods

### 2.1. Study Design and Participants

In this study, 786 adults aged 18 to over 50 participated. All the participants signed a consent form online and agreed to participate in the study. This cross-sectional survey was conducted on a sample of residents in SA from 10 August to 9 October 2021, corresponding with approximately 1 month of using an online electronic survey. The selection criteria were male or female Saudi citizens and residents over 18. Random convenience sampling and the snowball technique were used to collect data to reach all members of society. Participants were asked to complete an anonymous e-questionnaire created via Google Forms and distributed through various social media and connection platforms such as WhatsApp, Twitter, and email.

This study aimed to reach 1000 male and female participants in all regions of SA. The exclusion criterion for this study was that participants under 18 years were excluded. Ethical approval was obtained from the Ethics Research Committee at Taif University (KSA: HAP-02-T-105). All participants received a declaration of consent at the start of the online survey. Then, informed consent was obtained from all respondents. All methods were carried out in accordance with relevant Saudi guidelines and regulations.

### 2.2. Questionnaire

The questionnaire included a cover letter in Arabic and English, consisting of demographic and social information, general awareness about the pandemic, and statements in Likert scale format. It was designed to reflect the study population’s dietary habits and lifestyle during home isolation during the COVID-19 pandemic. The questions were developed based on a review of the literature [18]. Experts in the related field reviewed the questionnaire. Several revisions were made to strengthen the reliability and validity of the questionnaire and enhance the scientific value of the data to be collected. Consequently, a pilot study (*n* = 50 participants) was performed to confirm the reliability and validity of the questionnaire and obtained Cronbach’s α, which was noted to be excellent (overall 82%, demographics 84%, physical activity 85%, and dietary habits 86%).

The questionnaire began with an introduction describing the study’s aim and requesting consent from the participants. This was followed by 44 questions divided into three parts. The first part inquired about background information, including age, sex, height, weight, region of residence, education, work, monthly income, and marital status. The second part contained questions regarding health, mood, sleep, physical activity, and sedentary-behavior parameters such as screen time, relaxing activity, and desk-work time. In the final part of the questionnaire, the questions focused on dietary habits and supplement use during the isolation period; participants were asked to report the frequencies with which they eat fruits, vegetables, meat, fish, meals, and snacks.

### 2.3. Sample-Size Calculation

The sample size was calculated using Raosoft online calculator (Raosoft Inc., Seattle, WA, USA, http://www.raosoft.com/samplesize.html, accessed on 6 May 2020) to identify the number of participants required for this study. The Saudi population is approximately 34.22 million (SA Statistics Bureau, 2020, https://www.stats.gov.sa/en, accessed on 5 May 2020). The minimum sample size required for this study to achieve a 95% confidence interval and a 5% margin of error was 385 participants. However, in this study, the sample size was 786 to minimize errors and acquire more accurate results.

### 2.4. Data Analysis

Analyses were performed using the SPSS statistical package (IBM, Chicago, IL, USA). Microsoft Excel was used to illustrate the data. The nominal results are represented by proportions (%) and frequency (N), while the numerical results are represented by the mean and standard deviation (SD). A chi-squared test was used to detect any significant differences in categorical variables, based on our hypothesis that the COVID-19 pandemic may have triggered several changes in dietary habits, lifestyle, and physical inactivity among residents of SA. A *p*-value of <0.05 was considered statistically significant for all statistical tests.

## 3. Results

A total of 786 individuals participated in the current study. Table 1 depicts the socio-demographic profile of the participants, in which 92 (11.7%) were male and 694 (88.3%) were female. The mean age of participants was 30.48 ± 11.50. The majority (647; 82.3%) were from the Makkah region, while a minority of participants came from other regions of SA. Regarding marital status, 317 (40.3%) of the participants were married, 31 (3.9%) were divorced, 9 (1.1%) were widowed, and 429 (54.6%) were single. The mean number of family members was 5.84 ± 2.4. Most of the participants, 592 (75.3%), had a university education, while 52 (6.6%) had a master’s degree, 25 (3.2%) had a Ph.D., 99 (12.6%) had only a high school education, and a minority had education less than high school. As for the participants’ work status, 348 (44.3%) were students, 153 (19.5%) were working in the governmental sector, 61 (7.8%) were working in the private sector, 20 (2.5%) owned a business, 12 (1.5%) owned a business and held another job, 164 (20.9%) were not working, 23 (2.9%) were retired, and 5 (0.6%) had lost their jobs due to the quarantine. Their living situations were as follows: 307 (39.1%) lived in a villa, 441 (56.1%) lived in an apartment, and 38 (4.8%) lived in a traditional house; 259 (33%) were renting, while 527 (67%) owned their home. The mean monthly income of the participants was 14,520.5 ± 25,354.7.

### 3.1. Medical Profile of the Participants

**Figure 1:**
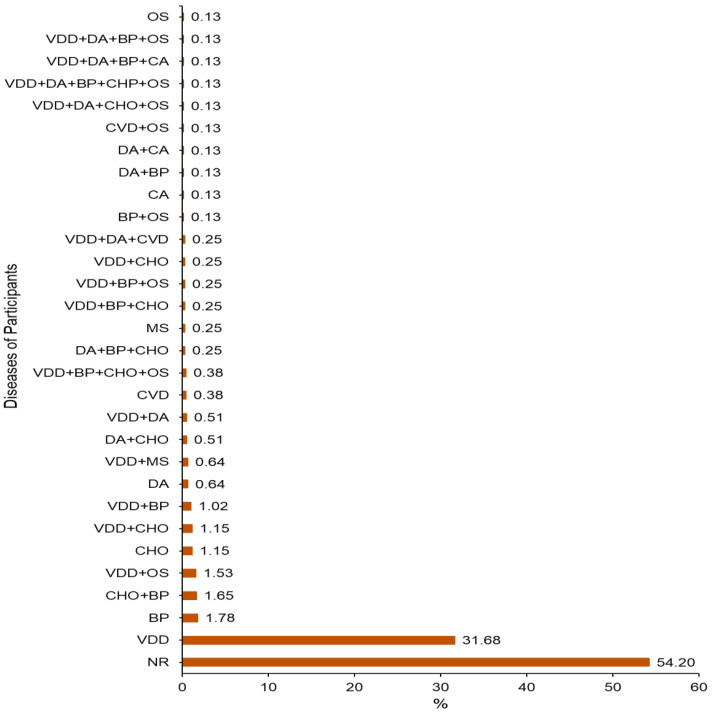
The medical profile of the participants, NR: Normal participants, VDD: Vitamin D deficiency, BP: Blood pressure, CHO: Cholesterol, OS: Osteoporosis, DA: Diabetes, MS: Metabolic syndrome, CVD: Cardiovascular disease, CA: Cancer.

Figure 1 gives the medical profile of the participants: 426 (54.20%) of the participants were normal, 249 (31.68%) had a vitamin D deficiency (VDD), 14 (1.78%) had hypertension (BP), 13 (1.56%) had BP and high cholesterol (CHO), 12 (1.53%) had VDD, 9 (1.15%) had CHO, 9 (1.15%) had VVD and CHO, 8 (1.02%) had VVD and BP, 5 (0.64%) had diabetes (DA), the same participants had VDD, and metabolic syndrome (MS), 4 (0.51%) had DA with CHO, 4 (0.51%) had DA with VDD, and 3 (0.25%) and 3 (0.25%) had VDD, BP, CHO, and Os. However, 1one to two participants (0.13 to 0.25) have been recorded with different and combined diseases. The incidence of COVID-19 among the participants was investigated. The participants were asked if they had COVID-19 before; 62 (7.9%) participants reported being previously infected with COVID-19, while 724 (92.1%) reported no previous infection.

### 3.2. The Participants’ Daily Routines, Mood, Sleeping, Physical Activity, and Smoking Habits during the COVID-19 Pandemic

Table 2 depicts the participants’ daily routines, mood, sleeping, physical activity, and smoking habits during the COVID-19 pandemic. They were asked if their daily routine had changed; 297 (37.79%) participants reported that it positively changed, 308 (39.19%) reported that it did not change, and 181 (23.03%) reported that it negatively changed. As for their mood, 118 (15.01%) participants reported always feeling positive, 400 (50.89%) reported tending to feel positive, 241 (30.66%) reported tending to feel negative, and 27 (3.44) reported always feeling negative. When asked if their sleeping pattern changed during the lockdown period, 204 (25.95%) stated that it improved, 354 (45.04%) reported no change, and 228 (29.01%) stated that it worsened. Regarding physical activity, 203 (25.83%) reported no working out during the quarantine, 390 (49.62%) reported working out irregularly, 58 (7.38%) reported working out regularly and weekly, and 135 (17.18%) reported working out periodically and daily; 476 (60.56%) described themselves as active before the pandemic, while 335 (42.62%) described themselves as active during the pandemic. In total, 146 (18.58%) participants were smokers; 47 (5.98%) reported a decline in their smoking rate during the pandemic, 61 (7.76%) reported the same smoking rate during the pandemic, and 38 (4.83%) reported an increased smoking rate during the pandemic.

### 3.3. Participants’ Dietary and Weight Changes during COVID-19 Pandemic

Table 3 illustrates the participants’ dietary and weight changes during the pandemic; 106 (13.5%) were underweight, 362 (46.1%) had a normal weight, 172 (21.9%) were overweight, 96 (12.2%) had class 1 obesity, 32 (4.1%) had class 2 obesity, and 18 (2.3%) had class 3 obesity. When asked about changes in the type of food they consumed during the pandemic, 313 (39.82%) reported a positive change, 338 (43%) reported no change and 135 (17.18%) reported a negative change. As for food they consumed during the pandemic, 247 (31.42%) reported an increase, 371 (47.2%) reported no difference, and 168 (21.37%) reported a decline in food consumption. When asked about weight changes during the pandemic, 229 (29.13%) reported weight gain during the pandemic, 287 (36.51%) reported no difference, and 270 (34.35%) reported weight loss during the pandemic. When asked if they had used supplements during the pandemic, 282 (35.88%) participants said yes, while 504 (64.12%) said no. The participants’ attitudes toward the consumption of fruits, vegetables, red meat, and fish are illustrated in Table 3; 75 (9.54%) reported increased fruit consumption, 67 (8.52%) reported increased vegetable consumption, 135 (17.18%) reported high red-meat consumption, and 238 (30.28%) reported increased fish consumption during the pandemic. The mean number of consumed meals per day during the pandemic was 2.73 ± 0.91, the mean number of consumed snacks per day during the pandemic was 2.2 ± 1.21, and the mean number of cups of water consumed per day during the pandemic was 5.76 ± 3.04.

### 3.4. Snack Preferences of Participants during COVID-19 Pandemic

**Figure 2:**
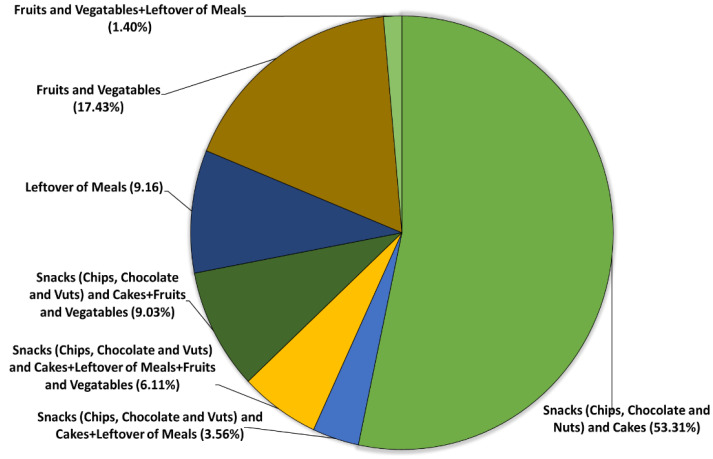
The snack preferences of participants during the COVID-19 pandemic.

Figure 2 depicts the snack preferences of participants during the pandemic; 419 (53.31%) preferred snacks (chips, chocolate, and nuts) and cakes; 28 (3.56%) consumed snacks (chips, chocolate, and nuts) and cakes with meal leftovers; 48 (6.11%) consumed snacks (chips, chocolate, and nuts) and cakes with meal leftovers and fruits and vegetables; 71 (9.03%) preferred snacks (chips, chocolate, and nuts) and cakes with fruits and vegetables; 72 (9.16) consumed only meals leftovers, while 137 (17.43%) preferred fruits and vegetables; whereas, only 11 (1.40%) consumed fruits and vegetables with meal leftovers.

### 3.5. Supplements Taken by the Participants during the COVID-19 Pandemic

Table 4 shows the supplements status of the participants during the pandemic; 504 (64.12%) reported taking no supplements, and 282 (35.88%) reported taking supplements. The detailed consumption of supplements is illustrated in the same table. Obviously, the majority of participants consumed multivitamins, vitamin C, and vitamin D: 48 (6.11%), 40 (5.09%), and 12 (1.53%), respectively. Moreover, many participants indicated mixed consumption of supplements of different kinds to increase their immunity.

### 3.6. Time of Participants Spent Sleeping, Doing Sedentary Activities, and Working out during COVID-19 Pandemic

Table 5 illustrates the time participants spent sleeping, doing sedentary activities, and working out during the pandemic. The mean number of sleeping hours during the pandemic was 8.19 ± 2. Watching TV accounted for a mean number of 4.23 ± 2.89 h, smartphone/tablet use accounted for 7.64 ± 5.02 h, computer/laptop use accounted for 3.00 ± 1.77 h, office work accounted for 3.16 ± 1.56 h, and resting and lying down accounted for 5.46 ± 5.44 h. The mean number of hours spent on social gatherings during the pandemic was 3.78 ± 3.72, while the mean number of minutes spent working out during the pandemic was 39.07 ± 22.84.

### 3.7. Gender-Based Comparison of Weight, Dietary Habits, Smoking, Working Out, and Sedentary Activities

Table 6 shows a gender-based comparison of the participants’ weight, dietary habits, smoking, working out, and sedentary activities. A significant difference between males and females was found regarding snack preferences for cake, chips, chocolates, and nuts (*p* = 0.022): more women preferred these snacks than men (73.3% vs. 62%). A significant difference was found between men and women regarding changes in vegetable consumption during the pandemic (*p* = 0.004). Men and women had a significant difference regarding fish consumption during the pandemic (*p* = 0.02). A significant association was also found between gender and using supplements during the pandemic (*p* = 0.037): a higher rate of supplement use was noticed among females than males (37.2% vs. 26.1%). A significant difference was found in smoking rates between men and women (*p* < 0.001), with men having higher smoking rates than women (45.7% vs. 15%). A significant difference between men and women was apparent when comparing hours spent watching TV (*p* = 0.035), hours spent on smartphones and tablets (*p* = 0.028), and hours spent on computers and laptops (*p* = 0.038). Women spent more hours on smartphones and tablets, while men spent more hours on TV, computers, and laptops.

## 4. Discussion

This study provides a clear picture of the changes in dietary habits and lifestyle behaviors in a sample of 786 Saudi residents during the COVID-19 pandemic. The participants completed the electronic questionnaire in August 2020, roughly 6–7 weeks after the lockdown was imposed to prevent the further spread of the coronavirus. Many countries, including SA, have implemented social distancing measures to combat the spread of COVID-19. Due to the rules imposed, the lockdown had the positive effect of reducing the epidemic level. However, the fear of disease and death and these restrictions increased individuals’ stress loads and forced them to change their habitual behaviors [12,13,19,20,21].

The results show that 29% of participants reported weight gain during the lockdown. Similarly, other studies have reported increased weight gain during lockdown periods [19,20,22]. Due to the fear of coronavirus, people tended to amply supply their kitchens with various foods to avoid trips to grocery stores; this may have led to overconsumption of food [23], particularly canned long-shelf-life foods, which are often high in calories. Considering that the lockdown forced people to work from home, gyms and parks were closed, and many people stopped performing their normal daily routines, weight gain is a logical outcome of this general decrease in physical activity and energy outflow [21,22]. The study found that more than half of the participants reported decreased physical-activity levels, which is consistent with the findings of previous studies [19,24]; this was accompanied by an increase in mean sitting or lying down hours, which may also increase the risk of obesity [25] and mortality [26]. In addition, a significant neuropsychiatric burden can result from the sedentary lifestyle encouraged by lockdowns [20]. In addition, the fear of COVID-19 may lead to emotionally driven eating and cravings for foods rich in carbohydrates and fat [27].

Furthermore, studies have shown that regular physical activity may improve the immune response to respiratory infections and decrease the risk of COVID-19 [28,29]. These findings notwithstanding, 36.51% of respondents reported no change in weight. This could be attributed to their level of awareness and education, or perhaps they were simply unaffected by quarantine [11]. Results of our study indicate that—in contrast to the Health Ministry guidelines—individuals changed their eating practices, with overconsumption of poor-quality foods and more snacks between meals, often consisting of cakes and chips. Furthermore, while the recommended intake of fruit and vegetables is at least five servings daily (USDA 2015), which is especially important for supporting the immune system during a pandemic [30], our results show that intake of these food groups was lower during the pandemic. This finding is consistent with other studies reporting low fruit and vegetable consumption among adults in Saudi Arabia [22,31]. Moreover, studies conducted in the UK, Italy, and Poland have reported similar findings [20,21], likewise for the Middle East and North Africa [14]. In addition, pandemic lockdowns may have reduced the availability of fruits and vegetables and restricted the opening hours of grocery stores, which may have reduced fruit and vegetable consumption [19,21].

When comparing food preferences according to gender, it has been found that women tend to eat much more fruit and fatty food than men [32], which is consistent with the results of our study. This may be attributed to the increased stress levels that people may have experienced during lockdowns, which may have increased their appetites for empty calories, high-fat snack foods, and sweets [33].

Interestingly, supplement intake was higher among our respondents, especially for Vitamins C and D and multivitamins [10,11]. Supplement use was higher among women than men. A logical explanation is that fear of COVID-19 caused people to follow social media applications; since most of these users are women, they were more likely to hear suggestions that these vitamins are known to boost the immune system [27]. Thus, women may have been more likely to follow advice to increase their vitamins intake [34]. A previous study supports this finding and shows that around half of the study participants increased their supplement intake during the COVID-19 pandemic [22].

According to the General Directorate of Nutrition in SA, the recommended water intake is six cups. The current study indicates that water intake was high among the participants, which is considered to be a positive change, as dehydration may decrease mucosal immunity [35]. The result is similar to those reported in various studies [20,22,30]. Greater awareness of the benefits of proper water intake during quarantine, combined with easy access to water, may have led to this behavior [9].

### Strength and Limitation

Notably, this study aimed to identify the impact of quarantine on the diet habits and lifestyle patterns of the population of SA during the COVID-19 pandemic. The research results confirmed changes in behavior, which may help develop health and nutritional programs that enhance good behaviors and practices. This study has many limitations that limit its generalization. First, the non-randomized sampling method: information was collected 6–7 weeks after quarantine lockdown. Results should be interpreted cautiously, as most participants were females, and most of the data referred to one region of SA.

## 5. Conclusions

In conclusion, the pandemic triggered several changes in SA residents’ dietary habits, lifestyle behaviors, and physical activity levels. Weight gain, increased food consumption, and decreased most respondents reported physical activity. Observing changes in eating habits during the lockdown could aid in deciphering its ramifications for population health. Further studies using large, randomized, controlled trials covering multiple institutions are needed to observe the changes in dietary and lifestyle habits during the lockdown period, which may help elaborate on the pandemic’s consequences for population health and assist in formulating and implementing policies for future closures, supporting vulnerable groups. Undoubtedly, reliable health and nutrition information and psychological support remain critical during the pandemic.

## Figures and Tables

**Table 1:** Socio-demographic profile of the Saudi participants (*n* = 786).

Demographical Characteristics	*n*	%
**Gender**		
Male	92	11.7
Female	694	88.3
**Age** (Year, mean ± SD)	30.48 ± 11.50	
**Place of Residency**		
Makkah	647	82.30
Madinah	30	3.80
Tabuk	7	0.90
Baha	5	0.60
Asir	6	0.80
Jazan	9	1.10
Riyadh	39	5.00
Qasim	1	0.10
Hail	4	0.50
Al Jouf	1	0.10
Northern Borders	1	0.10
Eastern Region	36	4.60
**Marital Status**		
Married	317	40.30
Divorced	31	3.90
Widowed	9	1.10
Single	429	54.60
**Number of Family Members** (mean ± SD)	5.84 ± 2.40	
**Education**		
Uneducated	2	0.30
Primary School Education	4	0.50
Intermediate School Education	12	1.50
High School Education	99	12.60
University Education	592	75.30
Master’s	52	6.60
Ph.D.	25	3.20
**Job**		
Student	348	44.30
Governmental Sector	153	19.50
Private Sector	61	7.80
Own Business	20	2.50
Own Business and Other Jobs	12	1.50
Not Working	164	20.90
Retired	23	2.90
I Lost My Job Due to the COVID-19 Pandemic	5	0.60
**Living in**		
Villa	307	39.10
Apartment	441	56.10
Traditional House	38	4.80
**Ownership of Household**		
Renting	259	33.00
Own Home	527	67.00
**Monthly Income (SAR)** (mean ± SD)	25,354.7 ± 14,520.5	

SD: Standard Deviation, SAR: Saudi Riyals.

**Table 2:** Daily routine, mood, sleeping, physical activity, and smoking during the COVID-19 pandemic (*n* = 786).

Question		*n*	%
	**Daily Routine**		
**Q1/ Did your daily routine change during the quarantine?**	Positively Changed	297	37.79
	Did Not Change	308	39.19
	Negatively Changed	181	23.03
	**Mood**		
**Q2/ How would you describe your general state of mood during the quarantine?**	Always Positive	118	15.01
	Tend to Be Positive	400	50.89
	Tend to Be Negative	241	30.66
	Always Negative	27	3.44
	**Sleeping**		
**Q3/ Did your sleeping pattern/hours change during the quarantine?**	Got Better	204	25.95
	Did Not Change	354	45.04
	Got Worse	228	29.01
	**Working Out**		
**Q4/ Did you work out during the quarantine?**	Did Not Work Out	203	25.83
	Worked Out Irregularly	390	49.62
	Worked Out Regularly and Weekly	58	7.38
	Worked Out Regularly and Daily	135	17.18
**Q5/ Would you describe yourself as active before the pandemic?**	Yes	476	60.56
	No	310	39.44
**Q6/ Would you describe yourself as active during the pandemic?**	Yes	335	42.62
	No	451	57.38
	**Smoking**		
**Q7/ Are you a smoker?**	Yes	146	18.58
	No	640	81.42
**Q8/ Would you describe yourself as active before the pandemic?**	Decreased	47	5.98
	Same	61	7.76
	Increased	38	4.83
	Not Smoker	640	81.40

**Table 3:** Dietary and weight change during COVID-19 pandemic (*n* = 786).

Question	*n*	%
**Q1/ BMI Classes:**		
Underweight	106	13.5
Normal	362	46.1
Overweight	172	21.9
Obesity Class 1	96	12.2
Obesity Class 2	32	4.1
Obesity Class 3	18	2.3
**Q2/ Did the type of food you eat change during the pandemic?**		
Positive Change	313	39.82
Did Not Change	338	43.00
Negatively Changed	135	17.18
**Q3/ Did the amount of food you eat change during the pandemic?**		
Increased	247	31.42
Did Not Change	371	47.20
Decreased	168	21.37
**Q4/ Did your weight change during the pandemic?**		
Increased	229	29.13
Did Not Change	287	36.51
Decreased	270	34.35
**Q5/ Did you use nutritional supplements during the pandemic?**		
Yes	282	35.88
No	504	64.12
**Q6/ Did your consumption of fruits change during the pandemic?**		
Increased	75	9.54
Did Not Change	486	61.83
Decreased	225	28.63
**Q7/ Did your consumption of vegetables change during the pandemic?**		
Increased	67	8.52
Did Not Change	504	64.12
Decreased	215	27.35
**Q8/ Did your consumption of red meat change during the pandemic?**		
Increased	135	17.18
Did Not Change	512	65.14
Decreased	139	17.68
**Q9/ Did your consumption to fish change during the pandemic?**		
Increased	238	30.28
Did Not Change	472	60.05
Decreased	76	9.67
**Question**	**Mean ± SD**	
**Q10/ How many meals did you consume per day during the pandemic?**	2.73 ± 0.91	
**Q11/ How many snacks did you consume per day during the pandemic?**	2.2 ± 1.21	
**Q12/ How many water cups did you drink per day during the pandemic?**	5.76 ± 3.04	

**Table 4:** Taken supplements by the participants during the COVID-19 pandemic.

Participants	*n*	%
No Supplements	504	64.12
Supplements	282	35.88
Total	786	100.00
**Supplements details**	***n*** **(282)**	**% (35.88)**
Mvit	48	6.11
Vit.C	40	5.09
Vit.D	12	1.53
Vit.C + Vit.D	9	1.15
Vit.C + Vit.D + Fe	8	1.02
Vit.C + Vit.D + Zn	8	1.02
Mvit + Vit.C	7	0.89
Omega-3	7	0.89
Vit.C + Zn	7	0.89
Mvit + Vit.D	6	0.76
Vit.C + Zn + Fe	6	0.76
Mvit + Omega-3	5	0.64
Mvit + Vit.C + Omega-3	5	0.64
Vit.C + Vit.D + Omega-3 + Fe	5	0.64
Vit.D + Fe	5	0.64
Vit.C + Omega-3	4	0.51
Fe	3	0.38
Mvit + Vit.C + Vit.D	3	0.38
Vit.Bc + Vit.D	3	0.38
Vit.Bc + Vit.D + Zn + Fe	3	0.38
Vit.C + Fe	3	0.38
Vit.C + Omega-3 + Se + Zn	3	0.38
Vit.D + Omega-3 + Fe	3	0.38
Zn	3	0.38
Mvit + Vit.Bc + Vit.C + Vit.D	2	0.25
Mvit + Vit.Bc + Vit.C + Vit.D + Vit.E + Omega-3 + Zn	2	0.25
Mvit + Vit.Bc + Vit.C + Zn	2	0.25
Mvit + Vit.C + Omega-3 + Zn + Fe	2	0.25
Mvit + Vit.C + Vit.D + Omega-3	2	0.25
Mvit + Vit.C + Vit.D + Omega-3 + Fe	2	0.25
Mvit + Vit.C + Vit.D + Se + Zn	2	0.25
Mvit + Vit.C + Vit.D + Zn + Fe	2	0.25
Mvit + Vit.C + Zn	2	0.25
Mvit + Vit.D + Fe	2	0.25
Mvit + Vit.D + Omega-3	2	0.25
Mvit + Vit.D + Omega-3 + Fe	2	0.25
Mvit + Zn	2	0.25
Vit.Bc + Vit.C	2	0.25
Vit.Bc + Vit.D + Omega-3	2	0.25
Vit.C + Vit.D + Omega-3 + Zn	2	0.25
Vit.C + Vit.D + Zn + Fe	2	0.25
Mvit + Fe	1	0.13
Mvit + Vit.A + Vit.B + Vit.C + Vit.D + Vit.E	1	0.13
Mvit + Vit.A + Vit.B + Vit.C + Vit.D + Zn + Fe	1	0.13
Mvit + Vit.A + Vit.C + Vit.D + Omega-3 + Zn	1	0.13
Mvit + Vit.A + Vit.D + Omega-3	1	0.13
Mvit + Vit.Bc	1	0.13
Mvit + Vit.Bc + Vit.C + Omega-3 + Zn	1	0.13
Mvit + Vit.Bc + Vit.C + Vit.D + Zn + Fe	1	0.13
Mvit + Vit.Bc + Vit.C + Zn + Fe	1	0.13
Mvit + Vit.Bc + Vit.D	1	0.13
Mvit + Vit.Bc + Zn	1	0.13
Mvit + Vit.C + Fe	1	0.13
Mvit + Vit.C + Fe	1	0.13
Mvit + Vit.C + Omega-3 + Zn	1	0.13
Mvit + Vit.C + Vit.D + Fe	1	0.13
Mvit + Vit.C + Vit.D + Omega-3 + Zn + Fe	1	0.13
Mvit + Vit.C + Vit.D + Vit.E + Omega-3	1	0.13
Mvit + Vit.D + Se	1	0.13
Vit.A + Vit.B + Fe	1	0.13
Vit.A + Vit.B + Vit.C + Vit.D + Omega-3 + Fe	1	0.13
Vit.A + Vit.B + Vit.C + Vit.D + Se	1	0.13
Vit.A + Vit.B + Vit.C + Vit.D + Vit.E + Omega-3 + Se + Zn + Fe	1	0.13
Vit.A + Vit.B + Vit.C + Vit.D + Vit.E + Omega-3 + Zn + Fe	1	0.13
Vit.A + Vit.B + Vit.C + Vit.D + Zn + Fe	1	0.13
Vit.A + Vit.B + Vit.D + Vit.E + Fe	1	0.13
Vit.A + Vit.B + Zn	1	0.13
Vit.A + Vit.C + Vit.D + Omega-3 + Fe	1	0.13
Vit.Bc + Vit.C + Omega-3	1	0.13
Vit.Bc + Vit.C + Omega-3 + Zn	1	0.13
Vit.Bc + Vit.C + Vit.D	1	0.13
Vit.Bc + Vit.C + Vit.D + Fe	1	0.13
Vit.Bc + Vit.C + Vit.D + Omega-3	1	0.13
Vit.Bc + Vit.C + Vit.E + Se + Zn	1	0.13
Vit.Bc + Vit.D + Se	1	0.13
Vit.C + Omega-3 + Fe	1	0.13
Vit.C + Omega-3 + Zn	1	0.13
Vit.C + Omega-3 + Zn + Fe	1	0.13
Vit.C + Vit.D + Omega-3	1	0.13
Vit.C + Vit.D + Omega-3 + Zn + Fe	1	0.13
Vit.D + Omega-3 + Zn	1	0.13
Vit.D + Zn + Fe	1	0.13
Zn + Fe	1	0.13

*n*: Participant number, Mvit: Multivitamins, Vit. C: Vitamin C, Vit.D: Vitamin D, Vit. A: Vitamin A, Vit.Bc: Vitamin B complex, Vit. E: Vitamin E, Omega-3: Omega-3 fatty acids, Fe: Iron, Zn: Zinc, Se: Selenium.

**Table 5:** Time spent on sleeping, sedentary activities, and working out (*n* = 786).

Question	Mean ± SD
**Sleeping**	
Q1/ How many hours do you sleep during the quarantine?	8.19 ± 2.00
**Sedentary Activities**	
Q2/ How many hours do you spend watching TV during the pandemic?	4.23 ± 2.89
Q3/ How many hours do you spend on your smartphone/tablet during the pandemic?	7.64 ± 5.02
Q4/ How many hours do you spend on your computer/laptop during the pandemic?	3.00 ± 1.77
Q5/ How many hours do you spend working in your office during the pandemic?	3.16 ± 1.56
Q6/ How many hours do you spend resting and lying down during the pandemic?	5.46 ± 5.44
Q7/ How many hours do you spend on social gatherings (either with the family or via phone/video calls) during the pandemic?	3.78 ± 3.72
**Working Out**	
Q8/ How many minutes do you work out per session during the quarantine?	39.07 ± 22.84

**Table 6:** Gender-based comparison of weight, dietary habits, smoking, working out, and sedentary activities.

Factor	Gender		*p*-Value
	Male	Female	
**Weight, Dietary Habits, and Smoking During The Quarantine**			
Did your weight change during the pandemic? (*n*, %)			0.069
Increased	20 (21.7%)	209 (30.1%)	
Did Not Change	31 (33.7%)	256 (36.9%)	
Decreased	41 (44.6%)	229 (33.0%)	
How many meals did you consume per day during the pandemic? (mean ± SD)	2.66 ± 1	2.73 ± 0.9	0.487
How many snacks did you consume per day during the pandemic? (mean ± SD)	2.09 ± 1.44	2.21 ± 1.18	0.349
Which of the following snacks did you prefer to consume during the quarantine? (*n*, %)			
Cake, Chips, Chocolates, and Nuts	57 (62%)	509 (73.3%)	0.022 *
Fruits and Vegetables	33 (35.9%)	234 (33.7%)	0.682
Meal Leftovers	21 (22.8%)	138 (19.9%)	0.509
Did your consumption of fruit change during the pandemic? (*n*, %)			0.114
Increased	14 (15.2%)	61 (8.8%)	
Did Not Change	56 (60.9%)	430 (62%)	
Decreased	22 (23.9%)	203 (29.3%)	
Did your consumption of vegetables change during the pandemic? (*n*, %)			0.004 *
Increased	11 (12%)	56 (8.1%)	
Did Not Change	69 (75%)	435 (62.7%)	
Decreased	12 (13%)	203 (29.3%)	
Did your consumption of red meat change during the pandemic? (*n*, %)			0.802
Increased	14 (15.2%)	121 (17.4%)	
Did Not Change	60 (65.2%)	452 (65.1%)	
Decreased	18 (19.6%)	121 (17.4%)	
Did your consumption of fish change during the pandemic? (*n*, %)			0.02 *
Increased	38 (41.3%)	200 (28.8%)	
Did Not Change	50 (54.3%)	422 (60.8%)	
Decreased	4 (4.3%)	72 (10.4%)	
Did you use nutritional supplements during the pandemic? (*n*, %)			0.037 *
Yes	24 (26.1%)	258 (37.2%)	
No	68 (73.9%)	436 (62.8%)	
Are you a smoker? (*n*, %)			<0.001 *
Yes	42 (45.7%)	104 (15%)	
No	50 (54.3%)	590 (85%)	
**Working Out and Sedentary Activities**			
Did you work out during quarantine? (*n*, %)			0.269
Did Not Work Out	20 (21.7%)	183 (26.4%)	
Worked Out But Irregularly	54 (58.7%)	336 (48.4%)	
Worked Out Regularly and Weekly	4 (4.3%)	54 (7.8%)	
Worked out Regularly and Daily	14 (15.2%)	121 (17.4%)	
How many hours do you spend watching TV during the pandemic?	3.76 ± 3.66	2.77 ± 4.29	0.035 *
How many hours do you spend on your smartphones/tablets during the pandemic?	6.55 ± 3.84	7.78 ± 5.14	0.028 *
How many hours do you spend on your computer/laptop during the pandemic?	2.38 ± 3.2	1.69 ± 2.97	0.038 *
How many hours do you spend working in your office during the pandemic?	2.09 ± 2.82	1.49 ± 3.20	0.091
How many hours do you spend resting and lying down during the pandemic?	5.99 ± 6.02	5.39 ± 5.36	0.324
How many hours do you spend on social gatherings (either with the family or via phone/video calls) during the pandemic?	3.15 ± 3.17	3.86 ± 3.78	0.082

* Significant at *p* 0.05.

## Data Availability

The corresponding author can provide all the data used in the present study upon reasonable request.
